# Effectiveness of a Soft Robotic Glove to Assist Hand Function in Stroke Patients: A Cross-Sectional Pilot Study

**DOI:** 10.1155/2022/3738219

**Published:** 2022-04-25

**Authors:** Wachirayongyot Thimabut, Pim Terachinda, Wasuwat Kitisomprayoonkul

**Affiliations:** ^1^International Program in Clinical Sciences, Faculty of Medicine, Chulalongkorn University, Bangkok, Thailand; ^2^Department of Rehabilitation Medicine, Faculty of Medicine, Chulalongkorn University, Bangkok, Thailand

## Abstract

**Purpose:**

Stroke patients have difficulty performing tasks using their paretic hands. There are limited data on the effects of using a soft robotic glove to assist with hand function. The objective of this study was to investigate the effectiveness of a soft robotic glove in assisting hand function in stroke patients.

**Methods:**

This study was a cross-sectional pilot study. Twenty stroke patients with partial or complete hand weakness were recruited from a rehabilitation centre. The Box and Block Test (BBT) and the Action Research Arm Test (ARAT) were performed under two conditions: with and without use of the soft robotic glove. The order of the conditions was randomly assigned by a computer-generated program.

**Results:**

BBT scores increased 6.4 blocks when using the soft robotic glove (*p* < 0.001). ARAT grasp, grip, pinch, and overall scores increased by 27.08% (*p* < 0.01), 28.75% (*p* < 0.001), 15.89% (*p* < 0.01), and 21.15% (*p* < 0.001), respectively, using the glove versus not using the glove.

**Conclusions:**

The findings of this study suggest that using a soft robotic glove can assist a poststroke paretic hand in executing grasp, grip, and pinch.

## 1. Introduction

Stroke incidence has increased worldwide resulting in death and disability [1]. Loss of independence and functional ability occurs in many stroke survivors [2], and sequelae persist affecting hand function and activities of daily living (ADL) [3]. Robotic rehabilitation technology such as training equipment and assistive devices are currently available on the market. There are several studies of robotic devices for upper extremity training in stroke patients [4–10]. A soft robotic glove was also studied as an assistive device [11–15]. In recent years, various wearable hand robots for assisting hand function have been developed. Lightweight, low-cost exoskeletons, and soft robotic gloves were developed for poststroke hand rehabilitation [16, 17]. A single case study revealed that using a soft robotic glove increased Box and Block Test (BBT) scores in a muscular dystrophy patient [18]. Furthermore, new technology helps reduce costs and spur improvements in the manufacture of soft robotic gloves [19, 20]. A review indicated that actuator design, safety, and implementation are important considerations in the development of robotic devices [21].

In clinical testing, recovery of upper extremity function was acquired after 20 sessions of soft robotic hand training in chronic stroke patients [10]. Chronic stroke patients with impaired hand function gave positive feedback for a soft robotic glove system in functional tasks [13]. Stroke patients with severe hand impairment gained enhanced grip strength while using a soft robotic glove [15]. Stroke and multiple sclerosis patients noticed tight and sustained gripping while using a soft robotic glove [22].

In this study, we developed a low-cost soft robotic glove and aimed to investigate its effectiveness in assisting hand function in subacute and chronic stroke patients with partial or complete hand weakness.

## 2. Methods

### 2.1. Subjects

Twenty stroke patients were recruited from the Thai Red Cross Rehabilitation Centre. The study was registered at http://www.clinicaltrials.in.th (TCTR20190422003). This study protocol was approved by the Institutional Review Board, Faculty of Medicine, Chulalongkorn University (IRB No. 646/61, COA No. 227/2019).

Inclusion criteria included the following: (1) aged 18-80 years; (2) hemorrhagic or ischaemic stroke; (3) stable vital and neurological signs; (4) motor power of proximal upper extremity ≥3 with ability to reach out and motor power of hand <3, grading by the Medical Research Council (MRC); (5) sufficient cognitive and language abilities to follow instructions; and (6) ability to sit for at least 60 minutes.

Subjects with the following conditions were excluded: (1) musculoskeletal problems such as severe pain in any joints of the paretic upper extremity; (2) joint instability in the affected wrist and/or hand; (3) cognitive impairment (Thai Mental State Examination Scores ≤23); (4) contracture of the shoulder, elbow, wrist, or finger joint that hindered using a soft robotic glove; (5) severe hand spasticity (Modified Ashworth Scale (MAS) > 2; (6) ataxia of paretic upper extremity; and (7) allergy to soft robotic glove material.

### 2.2. Study Design

**Figure 1:**
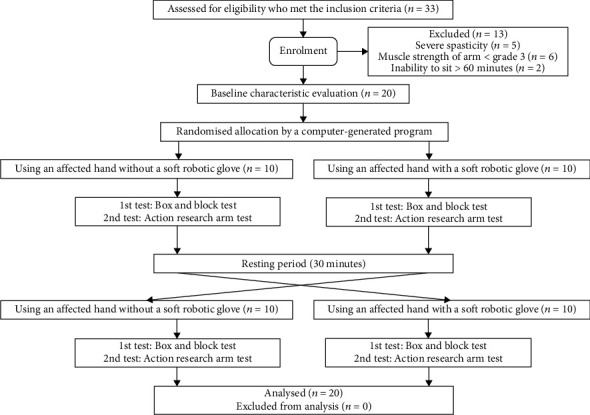
CONSORT flow diagram.

This study was a cross-sectional pilot study. There were two experiments: using an affected hand with the soft robotic glove and without the use of the soft robotic glove. The BBT and the Action Research Arm Test (ARAT) were performed by using the affected hand with and without the soft robotic glove in crossover experiments. Sequence of the experiments was randomly assigned by a computer-generated program. An occupational therapist opened a concealed envelope and supervised each patient completing an experiment following an allocation sequence. The CONSORT flow diagram is shown in Figure 1.

### 2.3. A Soft Robotic Glove

**Figure 2:**
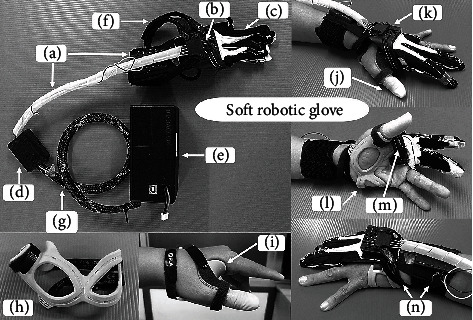
A soft robotic glove (a) hoist and cable system, (b) cable housing, (c) textile glove, (d) hand control switch, (e) power supply battery box, (f) wrist straps, (g) electric wire, (h) C-bar splint, (i) C-bar splint on the thumb, (j) latex glove on the thumb, (k) cable housing on dorsal side, (l) C-bar splint on the hand, (m) cable housing on palmar side, and (n) wrist straps on the hand.

A soft robotic glove has been developed by the Department of Mechanical Engineering, Faculty of Engineering, and the Department of Rehabilitation Medicine, Faculty of Medicine, Chulalongkorn University, Thailand. The glove is comprised of a hoist and cable-driven robot to assist flexion-extension of fingers, a textile glove, a hand control switch, and a power supply battery box (see Figures 2(a)–2(e)). The glove is a two-fingered design covering the index and middle fingers. Before wearing the soft robotic glove, stroke patients wear a C–bar splint to stabilise their thumbs (see Figures 2(h) and 2(i)). The metacarpophalangeal (MCP) joint of thumb was fixed in a 50-degree flexion position [23] by a C-bar splint. The soft robotic glove has 1 degree of freedom (DOF), i.e., finger flexion and extension. The maximal degrees of flexion of the fingers are 52^o^ at the MCP joint, 80^o^ at the proximal interphalangeal (PIP) joint, and 75^o^ at the distal interphalangeal (DIP) joint. Opening and closing of the soft robotic glove is controlled by the hand control switch (see Figure 2(d)). While a patient is pressing and holding the switch, cables in a housing located on the palmar side run a pulley and generate tension from the tendons in the textile glove (see Figure 2(m)). A pulling movement then assists fingers to flex. Flexing stops when the switch is released. To extend the fingers, the switch is pressed once again to passively extend the fingers. The pulley draws the cables back to the opposite direction and the tendon tension loosens. Fingers can then passively extend to release the object. A direct current motor is used to generate torque-controlled motion. The hoist and cable system transmits grip force at the fingertips between 12-28 Newtons depending on the required power to grasp, grip, or pinch objects. A force-sensitive resistor sensor is used to measure and determine the power and grip force for the different gasp, grip, and pinch. Subjects had to wear a latex glove on their thumbs for increased friction while executing experiments (see Figure 2(j)).

### 2.4. Procedures

**Figure 3:**
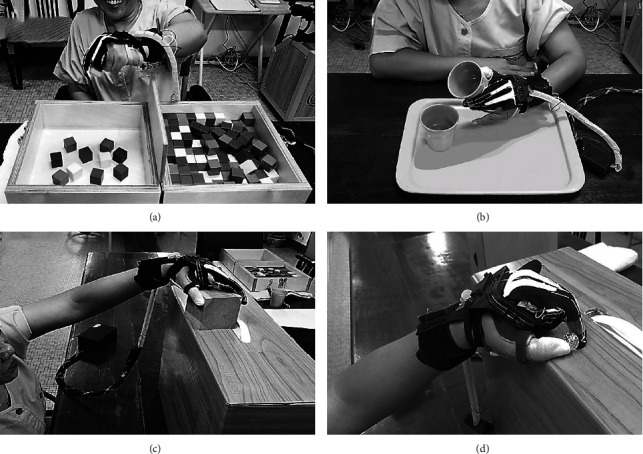
Subjects used the soft robotic glove to perform (a) the Box and Block Test, (b–d) the Action Research Arm Test: grasp, grip, and pinch.

Written informed consent was obtained from all subjects prior to participation in this study. Baseline characteristics were assessed: muscle strength; range of motion at the shoulder, elbow, wrist, and fingers; sensation of upper extremities; Brunnstrom's stages (BS); MAS of elbow, wrist, and finger flexors; and the Barthel Index (BI). The subjects were asked to perform the BBT and the ARAT in 2 experiments: using the affected hand with and without the soft robotic glove (see Figure 3). In each experiment, evaluation of the BBT was conducted first, followed by the ARAT. The subjects took a rest for 30 minutes between experiments in order to minimise fatigue.

For the BBT [24, 25], the subjects were asked to move a wooden block from one compartment to the other. The maximum number of blocks moved within 60 seconds was scored. For this test, the subjects were advised that their fingertips must cross the partition when transferring the blocks, and that they were not required to pick up the blocks that might fall outside of the box. For the ARAT [26], the subjects' coordination, dexterity, and functioning were assessed on four subscales (grasp, grip, pinch, and gross movement). Scores were rated on a 4-point scale, ranging from 0 (no movement) to 3 (movement performed normally) for a maximum score of 57.

The subjects were assigned to use the soft robotic glove in the experiments under supervision of an investigator who was on standby throughout the experiments. An occupational therapist was also invited to score each experimental evaluation.

### 2.5. Statistical Analysis

Based on a previous study of Takahashi et al. [27], the sample size was calculated by using the Power and Sample Size Calculation program, Version 3.1.2 (2014) according to mean difference of 12.189, standard deviation of 9.8282, and 90% power with a 2-sided significance level of 0.05. Calculated sample size was 9, but we set number of subjects at 20 because we would like to perform subgroup analysis regarding the BS of hand.

Continuous data was reported as mean and median. Categorical data was reported as frequency and percentage. The paired *t*-test compared the BBT and the ARAT scores between gloved and nongloved conditions. In addition, we conducted subgroup analysis on the BS of hand ≤3, and >3. Statistical analysis was performed by using the IBM SPSS Statistics for Windows, Version 22.0 (IBM Corp., Armonk, NY), with statistical significance set at a two-tailed *p* < 0.05.

## 3. Results

Twenty stroke patients completed the study. Baseline characteristics are shown in Table 1.

Comparison of BBT and ARAT scores when using the soft robotic glove and without the glove are summarised in Table 2. Significant improvement was demonstrated for both BBT and ARAT scores using the soft robotic glove compared to not using the glove. The BBT scores increased approximately fourfold from 2.2 blocks to 8.6 blocks (*p* < 0.001). The ARAT scores of grasp, grip, pinch, and total increased by 27.08% (*p* < 0.01), 28.75% (*p* < 0.001), 15.89% (*p* < 0.01), and 21.15% (*p* < 0.001), respectively. The difference in the ARAT score of gross movement was not found statistically significant (*p* = 0.186).

For subgroup analysis of the BS of hand, the BBT and the ARAT scores are summarised in Table 3. In stroke patients with the BS ≤ 3, the results showed that using the soft robotic glove significantly assisted hand function when compared to not using the glove. The BBT scores increased from 1.81 to 5.88 blocks with use of the glove (*p* < 0.05). When using the glove, the ARAT scores of grasp, grip, pinch, and total ARAT score also increased by 34.78% (*p* < 0.01), 39.67% (*p* < 0.001), 24.67% (*p* < 0.01), and 26.31% (*p* < 0.001), respectively. The results from subjects who had the BS > 3 showed that the BBT scores significantly increased from 3.75 to 8.5 blocks when using the soft robotic glove (*p* < 0.05), but there was no significant change in the ARAT scores.

There were no adverse events reported during or after the experiments.

## 4. Discussion

Subacute and chronic stroke patients achieved significantly higher BBT scores when using the soft robotic glove than without the soft robotic glove. The mean difference was 6.4 blocks, which corresponded to the minimally clinical important difference (MCID) of the BBT (6 blocks) [28, 29]. We also found that the soft robotic glove significantly assisted hand function, although the score did not meet the six-point MCID of the ARAT [28, 29]. Our soft robotic glove was safe and effective in helping patients achieve positive outcomes when performing the ARAT. Although scores on the gross movement subscale of the ARAT (i.e., place the hand behind the head, place the hand on top of the head, and move the hand to the mouth [26]) were not significantly different between gloved and nongloved trials, this may be explained by the tasks required strength only in the proximal upper extremity. Our soft robotic glove was developed to assist only hand function, not for the proximal part of the upper limb. Hence, this subscale did not significantly improve.

In a study of using a Soft Extra Muscle (SEM) Glove (Robotic SEM™ Technology, Sweden) in ten chronic stroke patients with impaired grasp, grip, and pinch with normal or mild sensory impairments and independence in ADL, median BBT scores 20 blocks and median ARAT scores 33.5 points were demonstrated while using the glove [22]. The study reported only median scores on the BBT and the ARAT while using the SEM Glove and did not report severity of hand impairment or baseline characteristics on the BBT and the ARAT. Thus, we could not compare the BBT or the ARAT scores using the SEM Glove with our study results.

The Hand Extension Robot Orthosis (HERO) Grip Glove (Toronto Rehabilitation Institute, Canada) study demonstrated that stroke survivors with limited active finger extension could transfer an average of 2.9 blocks (button mode) and 3.3 blocks (automatic mode) on the BBT with the robot-assisted mode using tripod pinch grasp [15]. Our soft robotic glove helped patients to complete the BBT with an average of 8.6 blocks in subacute and chronic stroke survivors with partial or complete hand weakness. We think that the subjects achieved well on the BBT because they had MRC grading ≥3 in their proximal upper extremities. The tripod-like motion of our soft robotic glove might potentially assist gripping and pinching objects of various shapes and sizes. The glove could also assist patients on the BBT due to the swift operation of its hoist and cable system.

Regarding the mean difference in the BBT and the ARAT scores in subjects with the BS > 3, the findings demonstrated that mean difference of these scores increased when using our soft robotic glove, but there was no statistical significance because of a small sample size and some variation. Additionally, we found that (1) the mean difference on the BBT increased beyond the MCID of the BBT [28, 29], and (2) the mean difference of the ARAT total score and the ARAT pinch score increased more when compared to subjects with the BS ≤ 3. One explanation may be that subjects with the BS > 3 had decreased spasticity and could perform complex movement combinations to obtain higher scores on the BBT and the ARAT. Further investigation on this subgroup with a larger sample size is required to clarify this relationship.

The findings from a study using an electromyography-driven soft robotic hand in rehabilitation training which included subjects with mild and moderate spasticity found that the soft robotic hand might be more beneficial in patients with mild hand spasticity [10]. In our study, we also excluded patients with severe hand spasticity. We believed that our soft robotic glove would not be suitable for use in subjects with severe spasticity because its swift finger movement might aggravate spasticity. We did not investigate the effects of using our soft robotic glove in performing ADL tasks assessed by the BI and the Functional Independence Measure as well.

In subacute and chronic stroke patients, training with a soft robotic glove required 6–7 weeks to improve hand function [9, 10]. In our study, we did not test it in a continuous rehabilitation setting to determine whether it could improve hand function recovery. Further study on our soft robotic glove as a long-term training device is required.

Design of a soft robotic device must address concerns of the control unit, portability, safety, wearability, actuation, and the human-robot interface [20]. The control unit of our soft robotic glove is characterised by portability and safety. Our soft robotic glove weighs 42 g, making it less cumbersome to wear while performing ADL [30]. The 475 g controller and battery box are separate from the glove. Hoists and cables provide smooth motion for assisting finger movement. The cost of our soft robotic glove is about 150 USD but could decrease if produced on a large scale and with a cheaper method for molding a transmission power box instead of the 3D printing method we used. Our soft robotic glove is controlled by a single hand switch and assists flexion and extension of finger joints to improve hand function. A single DOF produced by actuators controls the MCP, PIP, and DIP joints of the index and middle fingers.

Given the two-finger design of our soft robotic glove, subjects had to wear a latex glove on their thumbs to increase friction during object manipulation. We also used a C-bar splint to stabilise the thumb and provide proper hand position [23]. Some subjects could don/doff a C-bar splint and a soft robotic glove by themselves and took no more than 5 minutes. Some subjects required an assistant to help don/doff the splint and glove. Subjects suggested we make a robotic glove with a covered thumb design like the Exo-Glove Poly (Biorobotics Laboratory, Seoul National University, Republic of Korea) [16], or a full hand design like the HERO Grip Glove [15] because they thought a covered thumb design could help them perform tasks more easily and a five-finger design was familiar to them. Therefore, our soft robotic glove will require design improvements: (1) developing a 5-finger design that sets the thumb in a functional position; (2) creating a wireless system; (3) tailoring glove size to fit each user; and (4) designing a glove to be worn more easily. Our soft robotic glove already features safety features, noninvasiveness, a lightweight design, portability, ease of use, and ease of maintenance at a relatively affordable cost.

We recommend our soft robotic glove for grasp/grip/pinch assistance, e.g., grasping/gripping a cylindrical/spherical object (such as a 600 ml bottle, 375 ml can, 250 ml glass, tennis ball, an orange, and an apple), carrying a handle bag weighing less than 700 g, pinching a straw, and holding a mobile phone.

We suggest further investigation on the improvement of hand function and motor skills after rehabilitation with our soft robotic glove. A set of repetitive rehabilitation training sessions with long-term and home-based usage would be helpful with greater evaluation. Specific ADL tasks could be performed for expanded outcome assessment.

## 5. Conclusions

This study demonstrated that our soft robotic glove could assist paretic hands in executing grasp, grip, and pinch functions among subacute and chronic stroke patients who have hand weakness but sufficient proximal upper extremity strength to reach out with their arms, especially stroke patients who have the BS of hand ≤3. Findings from this study can help further refine the robotic glove design to improve its functionality.

## Figures and Tables

**Table 1:** Baseline characteristics.

	*n* = 20
Age (years), mean ± SD	55.1 ± 15.0
Gender, *n* (%)	
Male	14 (70)
Onset (months), median (range)	11.50 (1 − 84)
Stroke type, *n* (%)	
Ischaemic	15 (75)
Hemorrhagic	5 (25)
Side of weakness, *n* (%)	
Right	11 (55)
Dominant hand, *n* (%)	
Right	18 (90)
Affected hand, *n* (%)	
Dominant hand	11 (55)
Brunnstrom's stage of an affected hand, *n* (%)	
Stage 1	8 (40)
Stage 2	7 (35)
Stage 3	1 (5)
Stage 4	2 (10)
Stage 5	2 (10)
Stage 6	0 (0)
Modified Ashworth Scale of finger flexors, *n* (%)	
0	8 (40)
1	8 (40)
1+	3 (15)
2	1 (5)
3	0 (0)
4	0 (0)
Barthel Index, mean ± SD	13.4 ± 4.5

SD: standard deviation.

**Table 2:** BBT and ARAT scores.

	Using soft robotic glove (*n* = 20)	Without soft robotic glove (*n* = 20)	*p* value	95% CI
BBT, mean ± SEM	8.6 ± 2.0	2.2 ± 0.6	< 0.001	3.2, 9.6
ARAT, mean ± SEM				
Total	25.20 ± 4.43	20.8 ± 4.87	< 0.001	2.7, 6.1
Grasp	9.15 ± 1.5	7.20 ± 1.7	< 0.01	0.9, 3.0
Grip	5.15 ± 1.0	4.0 ± 1.1	< 0.001	0.6, 1.7
Pinch	6.20 ± 1.5	5.35 ± 1.7	< 0.01	0.4, 1.3
Gross movement	4.70 ± 0.8	4.55 ± 0.8	0.186	-0.1, 0.4

BBT: Box and Block Test; ARAT: Action Research Arm Test; SEM: standard error of mean; CI: confidence interval.

**Table 3:** BBT and ARAT scores analysed by BS.

	Using soft robotic glove	Without soft robotic glove	*p* value	95% CI
BS ≤ 3 (*n* = 16)				
BBT, mean ± SEM	7.69 ± 2.42	1.81 ± 0.68	< 0.05	2.06, 9.69
ARAT, mean ± SEM				
Total	20.69 ± 4.59	16.38 ± 5.13	< 0.001	2.75, 5.88
Grasp	7.75 ± 1.62	5.75 ± 1.85	< 0.01	1.01, 3.0
Grip	4.19 ± 1.0	3.0 ± 1.09	< 0.001	0.63, 1.75
Pinch	4.75 ± 1.48	3.81 ± 1.61	< 0.01	0.44, 1.43
Gross movement	4.0 ± 0.75	3.81 ± 0.8	0.188	-0.01, 0.48
BS > 3 (*n* = 4)				
BBT, mean ± SEM	12.25 ± 2.46	3.75 ± 1.55	< 0.05	0.23, 16.77
ARAT, mean ± SEM				
Total	43.25 ± 8.0	38.5 ± 9.75	0.222	-5.09, 14.59
Grasp	14.75 ± 1.89	13.0 ± 3.32	0.391	-3.82, 7.32
Grip	9.0 ± 1.78	8.0 ± 2.61	0.391	-2.18, 4.18
Pinch	12.0 ± 3.83	10.0 ± 3.74	0.252	-2.50, 6.50
Gross movement	7.50^∗^ ± 1.50	7.50^∗^ ± 1.50	—	—

^∗^p value and 95% CI could not be computed because the SEM of the difference was 0. BS: Brunnstrom's stage; BBT: Box and Block Test; ARAT: Action Research Arm Test; SEM: standard error of mean; CI: confidence interval.

## Data Availability

Data can be available upon request.
